# How can nanotechnology help to combat COVID-19? Opportunities and urgent need

**DOI:** 10.1186/s12951-020-00685-4

**Published:** 2020-09-05

**Authors:** Estefânia V. R. Campos, Anderson E. S. Pereira, Jhones Luiz de Oliveira, Lucas Bragança Carvalho, Mariana Guilger-Casagrande, Renata de Lima, Leonardo Fernandes Fraceto

**Affiliations:** 1 Human and Natural Sciences Center, Federal University of ABC. Av. dos Estados, 5001. Bl. A, T3 Lab. 503-3. Bangú, Santo André, SP Brazil; 2 São Paulo State University–UNESP, Institute of Science and Technology, Sorocaba, SP Brazil; 3 Universidade de Sorocaba, Rodovia Raposo Tavares km 92,5, Sorocaba, São Paulo Brazil

**Keywords:** Coronavirus, SARS-CoV-2, Nanotechnology, Nanoparticles, Nanosensors, Nano-vaccines

## Abstract

Incidents of viral outbreaks have increased at an alarming rate over the past decades. The most recent human coronavirus known as COVID-19 (SARS-CoV-2) has already spread around the world and shown R_0_ values from 2.2 to 2.68. However, the ratio between mortality and number of infections seems to be lower in this case in comparison to other human coronaviruses (such as severe acute respiratory syndrome coronavirus (SARS-CoV) and Middle East respiratory syndrome coronavirus (MERS-CoV)). These outbreaks have tested the limits of healthcare systems and have posed serious questions about management using conventional therapies and diagnostic tools. In this regard, the use of nanotechnology offers new opportunities for the development of novel strategies in terms of prevention, diagnosis and treatment of COVID-19 and other viral infections. In this review, we discuss the use of nanotechnology for COVID-19 virus management by the development of nano-based materials, such as disinfectants, personal protective equipment, diagnostic systems and nanocarrier systems, for treatments and vaccine development, as well as the challenges and drawbacks that need addressing.
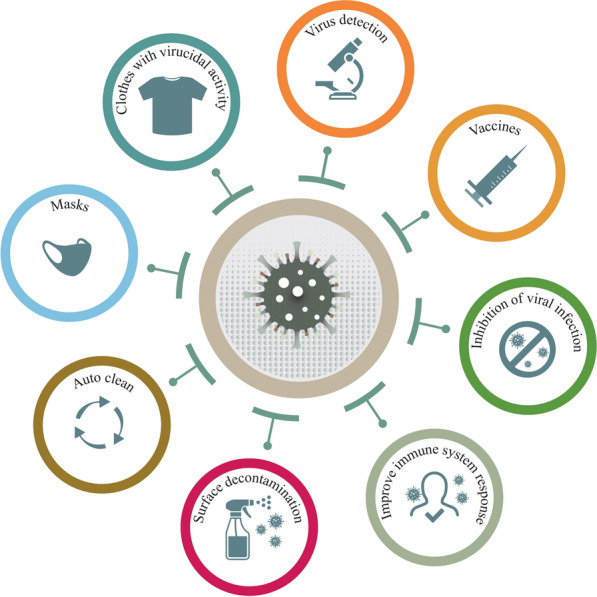

## Background

Coronaviruses belongs to the subfamily *Coronavirinae* (order: *Nidovirales*, family: *Coronaviridae*), which are enveloped and spherical viruses with a single-stranded RNA genome [1, 2]. The recent outbreak of the novel beta-coronavirus responsible for COVID-19 in Wuhan, China, is probably associated with a seafood market. According to WHO situation report 148, there had been 7,941,791 confirmed cases of COVID-19 globally by 16 June 2020, resulting in 434,796 deaths. According to Zhou et al., the genome sequence of the novel virus responsible for COVID-19 (denominated SARS-CoV-2) is 96.2% similar to the genome of bat coronavirus RaTG13, while it shares 79.5% similarity with SARS-CoV [3]. However, compared to SARS-CoV, the human to human transmission of SARS-CoV-2 is much faster, which has already resulted in its spread around the world [4–6] and led the WHO to declare the outbreak as a global pandemic on 11 March 2020 [7].

The genome sequence of SARS-CoV-2 shows that around two-thirds of the RNA is composed of replicase *ORF1a/1b*, which encodes 16 non-structural proteins and translates two polyproteins, followed by approximately 13 downstream ORFs. The rest of the viral genome encodes essential structural proteins that are spike (S), envelope (E), membrane (M) and nucleocapsid (N) [8–10]. This genomic organization is similar to bat-SL-CoVZC45, bat-SL-CoVZXC21, and SARS-CoV, as well as the length of most proteins encoded by these coronaviruses. However, a longer spike (S) protein is encoded by SARS-CoV-2 in comparison to bat SARS-like coronaviruses, SARS-CoV and MERS-CoV [9]. Recent studies have shown that novel SARS-CoV-2 and SARS-CoV infect host cells using the same angiotensin-converting enzyme 2 (ACE2) receptor [3, 11, 12]. The attachment of SARS-CoV-2 to the surface receptors of host cells are mediated by the S proteins [13]. Wrapp et al. showed the binding efficiency of SARS-CoV-2 to ACE2 receptors is 10- to 20-fold higher than that observed in SARS-CoV [13]. In addition, it was observed that cells that express ACE2, but not express the enzymes aminopeptidase N and human dipeptidyl peptidase-4, were more susceptible to SARS-CoV-2 entry [14].

Although the number of infected patients is continuously increasing, there are no officially approved drugs or vaccination for COVID-19 available yet. The current treatments are mainly based on symptomatic relief and respiratory support in seriously ill patients [15, 16]. Efforts to develop effective, targeted and safe drugs and vaccines to control this virus are currently underway. Some scientists have been looking into the similarity of transmission between the novel SARS-CoV-2 and SARS-CoV to develop drugs targeted towards highly conserved key proteins [17, 18], such as those involved in viral replication and proliferation. Examples of these proteins are spike, viral, and envelope proteins, as well as RNA proteases, which are specific viral targets. Host receptors and proteases, which are responsible for virus entry and endocytosis, are also potential targets for drug development [19–21]. Most of the currently available drugs for the treatment of viral infections fall in one of the followings classes: antiviral therapies, immune therapy, anti-inflammatory therapy, and other treatments that include traditional medicines based on natural products [22].

The effectiveness of conventional treatments of viral infections progressively fades away because of viral mutations and the resulting emergence of new viral strains [23]. Recently, the development of broad-spectrum antiviral drugs has caught the attention of researchers, as these drugs are less predisposed to resistance and could be used against several types of viruses, including new strains [24]. However, the development of new drugs is lagging behind the need for them because of the long process necessary to prove their efficacy and safety [25]. To overcome the limitations and to improve antiviral treatments, multidisciplinary research efforts are required toward the development of alternative antiviral therapies, targeting different phases in the viral replication cycle [26, 27]. In this regard, nanotechnology has attracted increasing attention and has already been investigated for potential use in prevention and/or treatment of viral infections [28–30].

Nanotechnology can be broadly defined as design and application of several materials and devices where at least one dimension is less than 100 nanometres. In the medical field, the application of nanotechnology is known as nanomedicine, which includes the use of nanomaterials for diagnosis, treatment, control and prevention of diseases [31, 32]. Over the decades, nanoparticles have been extensively used and studied due to their unique properties, such as small size, improved solubility, surface adaptability and multifunctionality, resulting in the development of better and safer drugs, tissue-targeted treatments, personalized nanomedicines and early diagnosis and prevention of diseases [33, 34]. Thus, it seems that nano-based approaches in the near future will be the first choice for the development of the most effective therapies for a wide range of diseases.

It is likely that nanotechnology holds huge potential in the diagnosis, treatment and prevention of COVID-19. Nanotechnology could help the fight against COVID-19 through different approaches, such as avoiding viral contamination and spray by: (a) design of infection-safe personal protective equipment (PPE) to enhance the safety of healthcare workers and development of effective antiviral disinfectants and surface coatings, which are able to inactivate the virus and prevent its spread; (b) design of highly specific and sensitive nano-based sensors to quickly identify the infection or immunological response; (c) development of new drugs, with enhanced activity, decreased toxicity and sustained release, as well as tissue-target, for example, to the lungs; and (d) development of a nano-based vaccination to boost humoral and cellular immune responses.

**Fig. 1:**
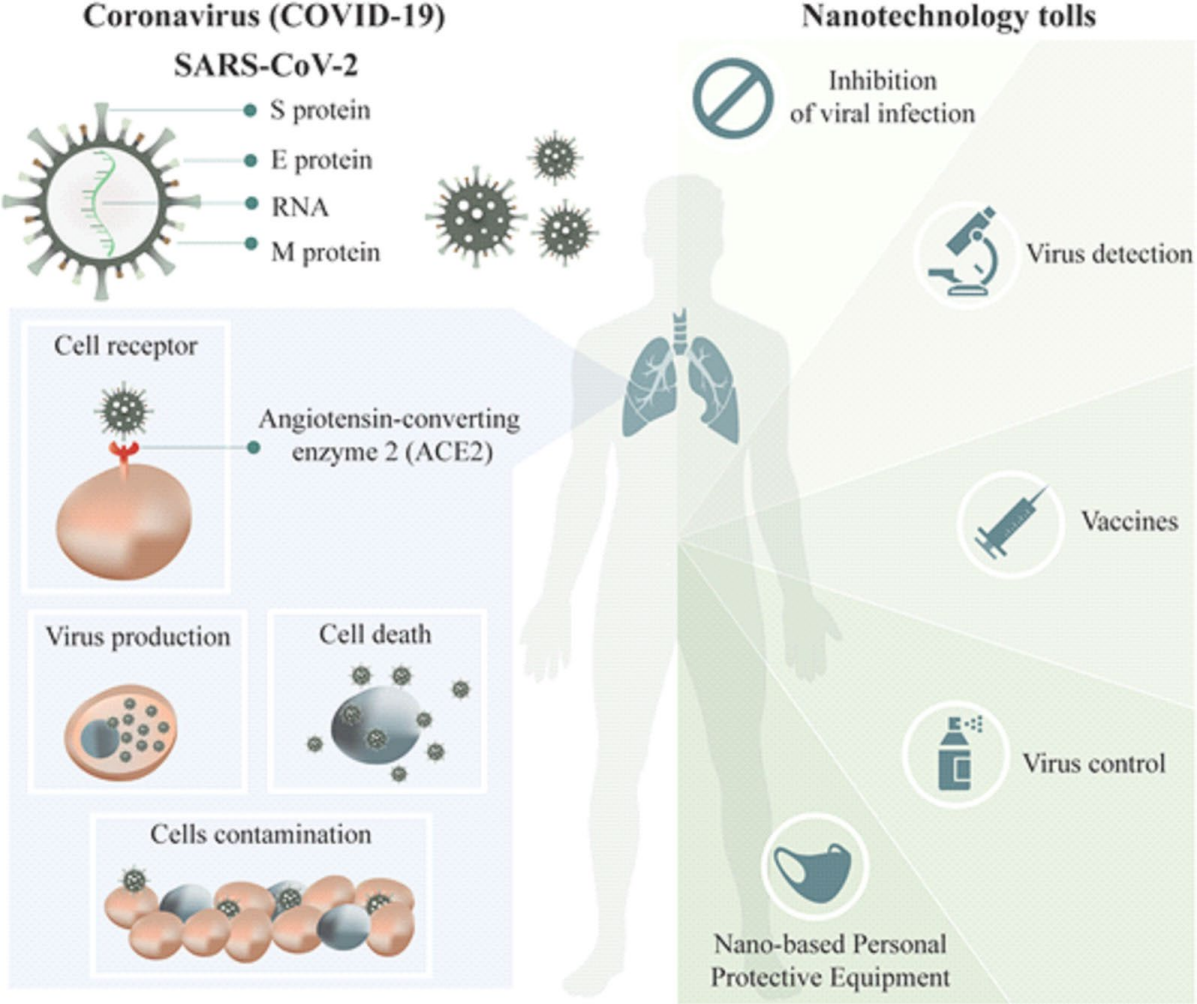
Schematic representation of SARS-CoV-2 infection and the nanotechnologies tools to prevent and control COVID-19. The virus entering into cell by the angiotensin-converting enzyme 2 (ACE2) receptor and use the host cell’s machinery to reproduce and contaminate new host cells. Nano-based materials could help in: (i) enhanced the speed and sensitivity of virus detection; (ii) help in the development of more efficient and safer treatment and vaccines and (iii) improve the safety of healthcare workers through the development of nano-based Personal Protective equipment (PPE)

Several nano-based formulations have been shown to improve the target delivery and therapeutic efficacy of antiviral drugs [28, 29]. In addition, due to the absence of therapeutic choices for various viral infections, many attempts have been done to explore the antiviral activity of natural compounds, such as plant metabolites [35]. However, most of the compounds found in plants have poor water solubility and low availability, resulting in a lack of therapeutic effect. In order to improve the therapeutic effect, botanical compounds have been combined with different nano-based carriers [36–38]. Moreover, nano-based biosensors could be used in diagnostics for the viral infection with high specificity and sensitivity [39, 40]. Another very promising approach is the new generation of vaccines based on different types of nanomaterials, with improved antigen stability, target delivery and controlled-release [41, 42]. Finally, the use of nanoparticle-based markers can enable the study of the mechanism by which viruses infect host cells [43–45] (Fig. 1).

Contaminated surfaces in public places, such as hospitals, parks, public transportation and schools, are a well-recognized common source for outbreaks of infection [46, 47]. Some studies have shown the potential of nano-based surface coatings for prevention of infections [48–50]. Also, the protection of healthcare workers is very important in a viral outbreak. This is where nano-based antimicrobial technologies can be incorporated into personal protective equipment for increased protection of healthcare workers [51] This review looks into the current approaches and advances in nano-based approaches for the control and treatment of COVID-19. The emphasis is on how nanotechnology can help fight viral infections and take account of any challenges in this regard.

## Nanotechnology strategies for disinfection of surfaces and PPE

COVID-19 is known to be very contagious and has many routes of transmission [52]. Recent studies have shown that SARS-CoV-2 spread through micro-droplets emitted mainly from person to person or through touching contaminated surfaces. The study by Van Doremalen et al. [53] showed that SARS-CoV-2 has the ability to persist for more than 3 h in aerosolized form. The study of Kampf et al. [54] indicated that, depending on the surface, the human coronavirus can persist for up to 9 days and at temperatures above 30 °C. It is in this context that the use of PPE, disinfectants and sanitizers is extremely important [55]. The World Health Organization (WHO) recommends the use of physical and chemical factors to mitigate contamination through the use of masks and hygiene personal care procedures, as well as disinfection of surfaces, especially for frequently touched surfaces, such as door handles, tables, chairs, handrails and switches. Different disinfectant agents are described in the literature, including sodium hypochlorite, hydrogen peroxide, alcohols, soaps/surfactants, etc. [56].

In a recent paper published by Huang et al. [57], authors described the prospects for researchers in the physical sciences and engineering fields to study these challenges and seek new solutions. Among the highlighted areas is the search for more efficient disinfectants and sanitizers. According to the authors, due to the different practical operations carried out, as well as volatilization processes, deparaffination, and degradation, water or alcohol-based disinfectants may not work completely and evenly on entire surfaces. Therefore, it is necessary to develop disinfectants and sanitizers that can last longer on surfaces, being resistant to constant washing and friction, in addition to presenting non-toxic properties.

### Nanomaterials for surface decontamination

This is where nanotechnology offers a lot of opportunities for the development of more efficient and promising disinfectant systems. Studies based on nanotechnology for the development of new materials, open perspectives for surfaces with self-cleaning properties [58]. These systems can have antimicrobial activity or be able to release chemical disinfectants slowly, increasing their time of action. Also, it can contribute to bringing in additional properties, such as responsive systems, that deliver active substances in response to different stimuli, such as photothermal, electrothermal, photocatalytic and others [59, 60]. Some metallic nanoparticles are also known to have a broad spectrum of action against viruses and other microorganisms [61]. Rai et al. [62] performed a literature review on the antibacterial, antifungal and antiviral potential of metallic nanoparticles. According to the authors, metallic nanoparticles, especially silver nanoparticles, could be used as a potent and broad-spectrum antiviral agent either with or without surface modification. However, the antiviral activity of these nanoparticles is still largely unexplored.

Vaze et al. [63] developed nano-disinfectants based on engineered water nanostructures (EWNS) generated through an electrospray and aqueous suspension ionization of different active ingredients. The results showed a significant reduction in pathogen concentration (including H1N1 influenza). In addition, the active ingredient (hydrogen peroxide) doses needed for inactivation were significantly lower (nanogram level), indicating the viability of this platform. The Nanotech Surface Company, for example, provides a disinfectant formulation based on titanium dioxide and silver nanoparticles. According to the company, the formulation allows the surfaces to be self-sterilized and was used recently during the COVID-19 pandemic for cleaning buildings in Milan [64]. Also, the company nanoSeptic developed a self-cleaning system for surfaces based on crystal nanoparticles, with one non-toxic system that did not generate residues. The nanoparticles promote an oxidation reaction process that is potentialized by light and acts against viruses or other microorganisms, keeping surfaces (such as door handles, elevator buttons and cell phones) clean [65].

Despite the advantages of these systems, they also have numerous challenges before they can be safely placed on the market. These challenges include scalability and production costs, issues of intellectual and regulatory properties and issues related to the potential toxicity and environmental effects of these systems [66]. Further studies are needed on the use of nanotechnology for more efficient disinfectant and sanitizing systems, as well as obtaining self-disinfecting surfaces to improve efficacy for infection control and health and environmental safety.

Table 1 shows published research and patents relating to different systems based on nanotechnology for application as disinfectants and sanitizers for viruses.

**Table 1:** Summary manuscripts found in the literature and patents related to disinfectants and sanitizers based on nanotechnology

Classification	Carrier system	Matrix	Properties	References
Article	NanoFilm	Polyvinyl alcohol (PVA) Polyolefin (POD) Sodium chlorite (NaClO_2_)	The authors obtained nanofilms containing NaClO_2_ crystals with the ability to release disinfectant gas (ClO_2_) after UV activation and exposure to moisture. According to the authors, the amount of gas released can be controlled by varying parameters, such as relative humidity, radiation dose, wavelength of UV radiation and activation mode	[67]
	Nanocomposite	Silica/silver	The authors successfully synthesized silica nanoparticles with silver in the core (10–20 nm) and in the crust (2–5 nm). According to the authors, nanoparticles containing silver from the centre have an advantage for slow and consistent release of Ag^+^ (confirmed in the tests). In addition, the prolonged release of silver occurred for more than 20 days	[68]
	Meso-structure nanoparticles	Electrically charged disinfectant (CAC-717)	According to the authors, treatment with CAC-717 allowed caused a reduction in the viral load to below the detection limit after 2 min of treatment. In addition, molecular biology assays have shown inhibition of both RNA and DNA nucleic acid amplifications, indicating that the disinfectant inactivates viruses and bacteria by modifying these molecules	[69]
	NanoStructure	Cellulose	The high alcohol content of hand sanitizer products currently on the market can cause skin dehydration. The authors address opportunities in the manuscript to develop innovative products based on nanocelluloses as vehicles for disinfectants and sanitizing agents	[70]
	Nanocomposite	Silica/silver	The authors obtained a new hybrid silica composite containing silver nanoparticles (Ag30-SiO_2_), which is approximately 400 nm in diameter. The Ag30-SiO_2_ particles showed an inhibitory effect against the dose-dependent influenza A virus (IFV-A). The results also suggested that the main antiviral mechanism of the systems was the interaction with the viral components located on the membrane	[71]
	Nanoparticles	Titanium dioxide	The aim of the study was to evaluate the effectiveness of titanium dioxide nanoparticles against microorganisms, including viruses found on different surfaces. According to the authors, there was a significant reduction in viral load under light and dark conditions, with an increase in effectiveness under light conditions. The authors also highlighted that the interaction with different surfaces influenced the result of disinfectant efficacy	[72]
	Photocatalytic nanostructured films	Titanium dioxide Silicon	The authors obtained nanostructured films based on silicone containing titanium nanoparticles with a high surface area (150 m^2^/g). The activity of nanofilms was investigated in combination with UV-A lighting (intensity of 22 W/m^2^), which is comparable to that of sunlight. According to the authors, after 20 min of exposure to the UV-A activated system, there was damage to microorganisms (bacteria and viruses) in addition to changes in the amount of fatty acids, indicating an interaction with the membrane	[73]
	Polyion complex nanoparticles (PCNs)	Poly[3-(acrylamido) propyl] trimethylammonium chloride (PAMPTMA)	The present study describes the potential of PCNs with the combination of anionic surfactants as a disinfectant for different microorganisms. The authors described that changes in hydrophobicity had little influence on the biological property of nanoparticles. A model bacterium (*Escherichia coli*) was used to assess the disinfectant power, and the results showed a rapid inhibitory effect (> 99.99% of death within 10 min of treatment)	[74]
	Biogenic nanoparticles	Iron Silver	The authors synthesized via FeG nanoparticles co-doped with Mn-Ag from the extract of *Solanum trilobatum* leaves. The characterization of the nanoparticles indicated iron particles in a spherical shape. Besides, it said the nanoparticles showed activity against pathogens (bacteria and viruses), being less toxic than their commercial analogue. According to the authors, the system can be used as a surface disinfectant	[75]
Patents	Polymeric nanoparticles	C1–C4 monohydric alcohols and different lipids	The invention relates to a germicidal composition for topical application (hands, arms, legs, face etc.). According to the inventors, the composition has high cleaning power due to the presence of a large amount of alcohol, in addition to solid lipid nanoparticles that increase effectiveness, controlling losses	[76]
		Poly(lactic-co-glycolic acid) (PLGA) essential oil	The invention describes obtaining a formulation based on poly(lactic-co-glycolic acid) (PLGA) nanoparticles containing essential oil (which may be of different origins). In addition to the preparation, the invention also provides the application of nanoparticles with a hand sanitizer. According to the inventors, the system has a high encapsulation rate, a good slow-release effect and effectively prevents oxidative damage to the essential oil	[77]
		Polyethylenimine (PEI) Polydiallyldialkylammonium salt Poly(acrylamide-co-diallyldialkylammonium halide) Chitosan	The invention provides an antimicrobial composition based on different polymers as carrier agents in order to mitigate the transmission of infectious diseases from surfaces. According to the inventors, the cartridges are water-soluble and non-toxic and can be composed of different sanitizing agents, also including inorganic particles	[78]
		Sulfonylalkylcyclodextrins	The invention relates to a composition of a viricidal formulation and its use in the treatment of viral infections, as well as for sterilization and disinfection. The composition is based on the activity of different alkyl sulphate groups and a cyclodextrin carrier. The inventors describe a high viricidal activity of the system, also showing a residual effect	[79]
	Biogenic nanoparticles	Silver	The invention describes obtaining a formulation based on silver nanoparticles obtained through an ecological route. According to investors, the environmentally friendly method that uses natural reducing agents presents a moderate reaction, short synthesis time and low production cost	[80]
	Photocatalytic systems	Tungsten trioxide Palladium	The present invention describes a formulation to disinfect surfaces and fluids using a photo-catalyst system. The system is based on tungsten trioxide nanoparticles doped with palladium nanoparticles (concentration of 0.1–5% of the total weight of tungsten trioxide nanoparticles). The authors describe the high disinfectant power of the system	[81]
		Tartaric acid Titanium isopropoxide (IV)	The invention relates to a method for preparing a self-decontamination surface. The authors describe that the method consists of dissolving tartaric acid in water and doping with titanium (IV) isopropoxide nanoparticles. According to the authors, the coating is fully mouldable and has a prolonged biocidal function	[82]
	Metal nanoparticles	Titanium dioxide Citric extracts	The present invention relates to obtaining a conjugated formulation of titanium dioxide nanoparticles and plant extracts (herbs and/or fruits). According to the inventors, the formulation is prepared by impregnating different functional groups of the extracts, which confer different properties (viricidal, bactericidal, fungicidal, mycobactericidal, etc.). Also, according to the inventors, the activity is dependent on the size of the nanoparticles, and the formulation is a liquid suspension	[83]
		Silver	The invention relates to a nanoparticle formulation preparation stabilized with different polymers. The inventors describe that these particles can be used for the disinfection of surfaces, and because of their greater stability, they are able to achieve more effective control over time	[84]
		Silver Quaternary ammonium salt	The invention describes a method for preparing a silver nanocomposite/quaternary ammonium salt molecule. The inventors describe different steps for preparing silver nanoparticles and mixing the with quaternary ammonium salt using sonication with the addition of surfactant. Also, according to the inventors, the formulation has a durable performance and high sterilization capacity	[85]

### Development of nanomaterials for PPE

According the United States Centers for Disease Control and Prevention (CDC), the main factors for the spread of COVID-19 is close contact (person-to-person) and respiratory droplets produced by infected persons [86]. The use of appropriate PPE, such as masks and gloves, is also important to combat the spread of the coronavirus. However, there are many issues regarding the availability and appropriateness of PPE products, for example facemasks not fitting properly or not suitable for restricting airborne viral particles [87, 88]. Nanotechnology is offering new materials that are more comfortable, resistant, and safer means for protection against biological and chemical risks [87, 89–91]. Facemasks, lab or medical aprons and others have been nanoengineered to provide new functions, for instance, hydrophobicity and antimicrobial activity without affecting the material’s texture or breathability [90, 91]. The hydrophobicity of PPE products can provide an effective barrier on its own against airborne droplets emitted during coughing or sneezing.

Examples of building hydrophobicity into textiles include the use of a billion tiny fibres, which are collectively called nanowhiskers, of hydrocarbons that are 3-fold smaller than a cotton fibre and increase surface tension, preventing absorption of droplets. Other methodologies include nanoscale 3D surfaces, structuring of materials and/or coating with hydrophobic nanoparticles [90, 92].

Similarly, the use of nanomaterials can build antimicrobial properties in textiles used in PPE. This strategy has been used to prevent the growth of microorganisms in clothes [90, 93]. The surfaces modified by nanoscale biocides, such as quaternary ammonium or quaternary phosphonium salts, polymers or peptides, can control microorganisms through oxidation of the microbial membrane [92, 94]. One of the best examples of how nanotechnology can improve personal protection is the production of facemasks. Traditional facemasks have a gap between the fibres, averaging 10–30 µm, that is inadequate for avoiding virus contact, and the reduction of this gap between the fibres cause a reduction of breath and increases of both temperature and pressure, making it uncomfortable for the user [89]. Many frontline healthcare workers have been suffering from skin damage due to the continuous use of facemasks [95]. The use of nanomaterials, such as nanofibers (composition not mentioned), can reduce breathing resistance and drop the pressure to provide wearing comfort, but at the same time protect against small particles (< 50 nm) [87]. This provides much better protection than traditional surgical facemasks, which do not offer protection against particles 10–80 nm in size. For example, N95/FFP2 facemasks can only protect against particles 100–300 nm in size [52]. Another strategy also used to increase the personal protection of facemasks is the modification of the textile surface. The use of nanoparticles, such as copper and silver, provide antimicrobial activity and can be included in different types of fibres or materials, such as cotton, polyalkene, polyester, polyamide, polyaramide and cellulose-based polymers [93, 96, 97]. Park et al. showed that silver nanoparticles (in the form of a silica hybrid composite) can be used in filters or membranes. The nanoparticles can inactivate influenza virus due to its interaction with the membrane of the virus [71]. Fudzhimori et al. have described a technology-based on monovalent copper compound and/or iodide fine nanoparticles for antimicrobial textile products. The system can inactivate influenza virus and has potential against different viruses, such as human coronavirus or SARS-CoV [98].

This use of nanomaterial for facemasks has two positive points. First, facemask protection works as a filter plus microbicidal agent, resulting in blocking and inactivating/killing the pathogens. Second, the management of this material after its use becomes safer once the biggest part of pathogens is destroyed in contact with the masks reducing the probability of contamination during the undressing process. The technology patents also leave this technology open for the production of other kinds of PPE, such as visitor aprons, surgical aprons, medical and lab coats, foot protection and bedsheets, that can further help in avoiding the spread of pathogens [96, 97].

**Fig. 2:**
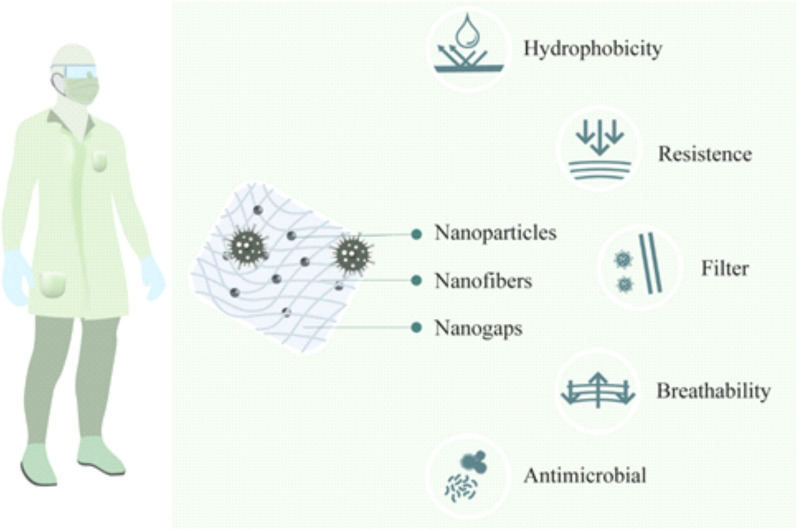
Nanotechnology applications for production of PPEs. The use of nanomaterials can give new properties making the materials more resistant, efficacious, comfortable and safer for use

Figure 2 shows the main advantages of nanotechnology applications for the production of PPE products.

For gloves, some products, based on silver nanoparticles, are available and are sold for their antibacterial effects. However, the system may also have a potential for virus protection, as studies have shown that silver nanoparticles also have viricidal activity [71, 91, 99].

The nanotechnology-based PPE can also be designed for multiple uses. Table 2 shows nano-based patents, products and applications for protection against viruses and other microbial pathogens.

**Table 2:** Patents that use nanomaterials for production of nano-based personal protective equipment (PPE) against microorganisms (i.e., virus, bacteria and fungi)

Nanomaterial	Purpose of application	References	
CeO_2_ micro- and nanoparticles	Protective topical treatments for skin protection or decontamination	[100]	
Electrospun polytetrafluoroethylene nanofibres	A filter capable of removing 99.999% of airborne particles with potential to be applied as respiratory protection	[101]	
Metal oxide nanoparticles (silver and copper)	Face masks with antimicrobial proprieties	[96]	
Nanofibres of polyvinylidene fluoride (PVDF) or nylon resin	Facemasks produced by nanofibres containing chlorhexidine gluconate or polyhexamethylene biguanide (PHMB) as an antimicrobial	[102]	
Nanofibres of polypropylene	Facemasks produced by nanofibres containing a pathogen collector and antimicrobial disposed of in one or more layers	[87]	
Antiviral mask (polyamidoamine)	Face masks with antiviral proprieties	[103]	
Nanofibres	Equipment that can be used for facemask protection	[88]	
Metallic nanoparticles	System for reduction and prevention of virus transmission by coating surfaces	[89]	
Copper and iodine nanoparticles	Virus inactive cloths (potential application for production of shoe covers, gowns, masks, gloves and filters)	[98]	
Polyester containing copper nanoparticles	Production of medical products, packaging paperboard, and cardboard	[97]	

Commercial products			
PPE	Product name	Purpose of application	Company
Masks	Surgical Masks-ESpin Technologies	Use of nanofibres for particles removal	ESpin Technologies, Inc.-USA
	Defenser Series-Respirator masks	The facemask has nanoparticles of silver and copper acting as a blend with antimicrobial activity	Nexera Medical-Canada
	The Guardian (valve)- reusable	The valve mask has nanoparticles of silver and copper acting as a blend with antimicrobial activity	Nexera Medical-Canada
	The Guardian masks- reusable	The valve mask has nanoparticles of silver and copper acting as a blend with antimicrobial activity	Nexera Medical-Canada
	MVX Nano Mask	A self-cleaning surgical mask containing titanium and silver zeolite nanoparticles	MVX Prime Ltd.
Gloves	Everyday Protect Gloves L	A product containing silver nanoparticles and the active compounds thiabendazole and zinc pyrithione	Mapa Spontex- United Kingdom
	PADYCARE^®^	Product coated with silver nanoparticles with antibacterial effect	TEXAMED^®^ GmbH-Germany
	Chlorhexidine wash gloves	A product containing silver nanoparticles and 2% chlorhexidine; the antibacterial effects last many hours after use	GAMA HEALTHCARE LTD.

Despite all the potential of nanomaterials for use in PPE products, the materials must also be evaluated for any side effects, such as skin irritation, allergy, or toxic effects, in humans. On the other hand, the nanoparticles can be released from clothes during the washing process, and they will eventually be released into the environment as waste. They may become a source of contamination, and therefore also need to be appropriately recycled to avoid negative environmental impacts.

## Strategies using nanotechnology for virus detection and disease diagnosis

Viruses are simple biological structures, size in the nanometre range (in the case of SARS-CoV-2, the size ranges from 60 to 140 nm) [104] and intracellular life cycle, often making their detection difficult, as they are difficult to be isolated and cannot be observed by ordinary optical microscopes [105, 106]. As with other viral infections, SARS-CoV-2 requires rapid response tests [64], with operational simplicity and better detection limits [107]. Therefore, detection and diagnosis is an important tool for the containment of COVID-19, as they contribute to the rapid implementation of control measures for the isolation and tracking of infections [108].

Specifically, when dealing with the detection and diagnosis of COVID-19, the tests are based on specific nucleic acids and proteins, as well as point-of-care testing [104]. Protein-based tests (serology) are a standard, widely accepted method as the first choice in large-scale tests for the detection of viruses in the body and are based on the presence of specific viral antigens or corresponding antibody responses of the immune system. However, the accuracy, reliability, and selectivity of these tests are commonly confronted by the possibility of cross-reactivity of the antibodies used, which can increase the risk of false positives [103]. Another important limitation occurs in the diagnosis of individuals with a viral load in its initial representation or when a mutation occurs during the propagation period [109].

All of these tests have their particularities and have positive points as well as negative points. Nanotechnology through its numerous applications is an efficient and cost-effective tool to be used to improve these tests for detection of SARS-CoV-2 [106]. A variety of nanomaterials, including metallic nanoparticles, polymeric nanoparticles, silica nanoparticles, carbon nanotubes, and quantum dots, are already used for virus detection [105, 110]. For the development of these systems for virus detection, the surface of the nanoparticle was modified with biomolecules derived from the virus, for example DNA, RNA, antibody, antigen (hemagglutinin antigen H1N1), peptide or pentabody (avian influenza virus–pVHH3B) [105, 110]. The high surface and volume ratios of nanomaterials improve the interactions between the sensor and the analyte, increasing the detection limit and decreasing the detection time [111].

In this context, nanotechnology-based probes have been widely used for the production of biosensors [106], in which the use of nanomaterials improves the sensor’s response, either through gaining electrical, optical or catalytical properties, providing greater analytical sensitivity for diagnosis [106, 112]. For the projection of virus detection tests, gold nanoparticles have stood out due to their photonic, electrical, and catalytic properties [105, 112, 113]. Specific thiolated probes, for example, functionalized with gold, can form disulphide bonds with complementary RNA from the target. It was this concept of a colorimetric assay that Kim et al. [113] used for detection of MERS-CoV. The gold nanoparticles were functionalized with probes modified with thiol on the surface, which hybridize with the target, preventing the aggregation of the nanoparticles by salts and consequently, the colour change, and this platform can be easily adapted for the diagnosis of other infectious diseases, such as COVID-19. Other tests incorporating gold nanoparticles combined with antibodies against SARS-CoV-2 IgG/IgM showed the potential for rapid symptomatic and asymptomatic screening for COVID-19 [114].

Transcription of the virus genomic sequence was one of the main steps for the development of sensors [104]. It is known that, among the SARS-related viral genomes, three regions have conserved sequences, which are the RNA-dependent RNA polymerase (RdRP) gene responsible for the ORF1ab region of the open reading frame, the envelope protein (E) gene and the nucleocapsid phosphoprotein (N) gene. These regions have been the focus of the primer and probe project, aiming at detection with considerable analytical sensitivity [109, 115]. The extraction of viral RNA is also the focus of the nanotechnology application, associating it with the use of molecular diagnostics already commonly applied. Research has shown that magnetic nanoparticles coated with silica can be used to quickly extract RNA from the virus in patient samples for later detection by RT-PCR [116, 117]. This cuts the needs for lengthy RNA extraction while also making the method more sensitive [117]. Another important point to be highlighted is the use of hybrid systems that combine the use of biomolecules derived from viruses with nanostructures, being an approach widely used in the development of sensors [105].

**Fig. 3:**
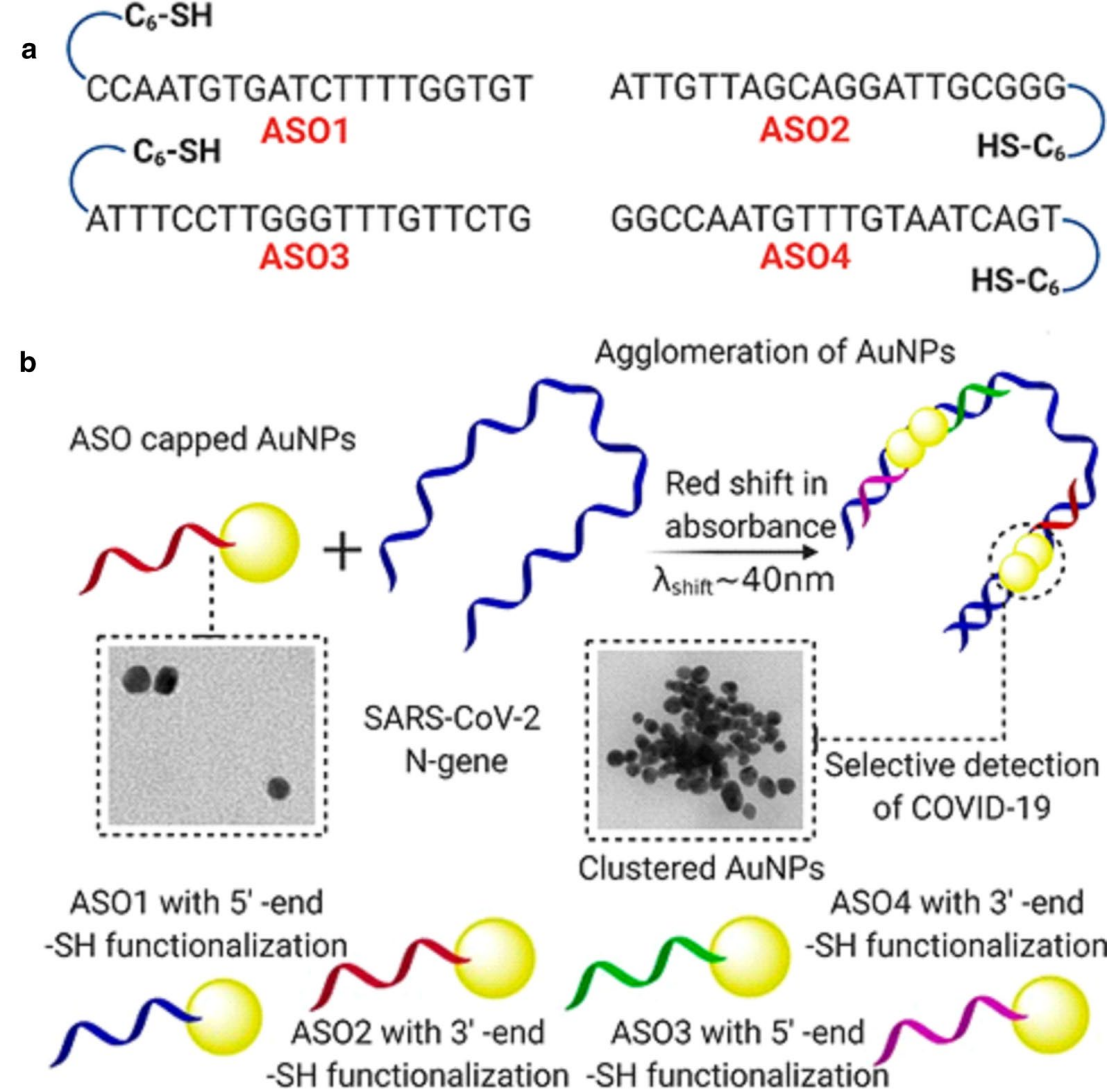
Differentially functionalized ASOs with their sequences are represented in a. The proposed concept behind the agglomeration of gold nanoparticles, when capped with the ASOs, is schematically presented in **b** (Reprinted with permission from Moitra et al. [109])

In this context of hybrid systems, Moitra and collaborators [109] reported the development of a selective assay that allows the detection of SARS-CoV-2 with the naked eye. The assay is based on gold nanoparticles coated with thiol-modified antisense oligonucleotides (ASOs), specific for the N gene of SARS-CoV-2, capable of diagnosing positive cases in 10 min. According to the study, changes in surface plasmon resonance observed using a UV–vis spectrophotometer indicate the selective agglomeration of coated nanoparticles in the presence of a SARS-CoV-2 target RNA sequence (Fig. 3). With the addition of RNAseH, the hybrid RNA chain breaks down, leading to the formation of a visually detectable precipitate.

**Fig. 4:**
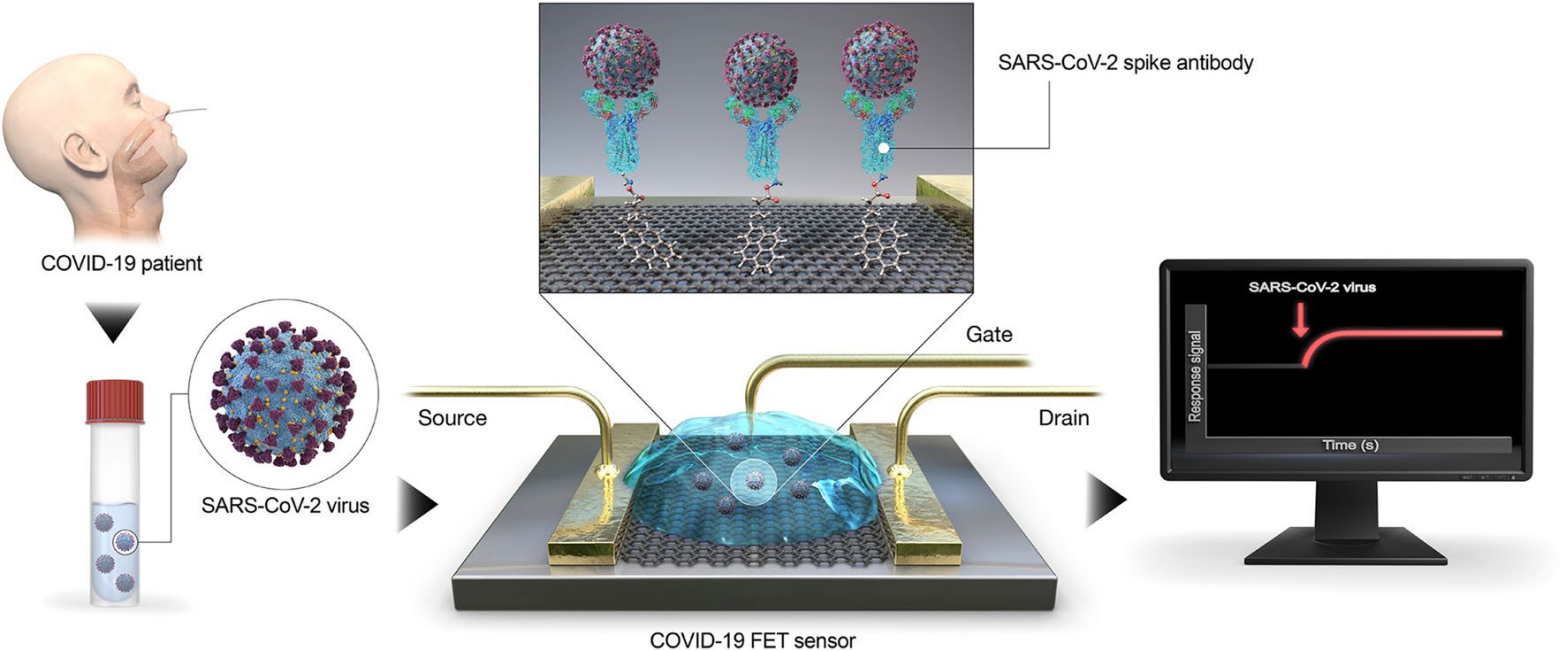
Schematic diagram of COVID-19 FET sensor operation procedure. Graphene as a sensing material is selected, and SARS-CoV-2 spike antibody is conjugated onto the graphene sheet via 1-pyrenebutyric acid N-hydroxysuccinimide ester, which is an interfacing molecule as a probe linker (Reprinted with permission from Seo et al. [118])

Another example is the graphene-based nanobiosensor reported by Seo et al. [118]. The authors developed the sensor to detect SARS-CoV-2 in clinical samples (Fig. 4). The sensor was produced using graphene nanosheets embedded with a specific antibody against the S protein of SARS-Co-V-2. Samples of antigenic protein, cultured virus, and a nasopharyngeal swab from patients with COVID-19 were used for system validation. The device was able to detect the S protein of SARS-CoV-2 in concentrations of 1 fg/mL in buffered saline. In addition, for clinical samples, a detection limit of 2.42 × 10^2^ copies/mL was reached. According to the authors, the advantage of the methodology is that, in addition to being highly sensitive, it does not require the pre-treatment of samples, since the sensor was able to detect the spike antigen protein of SARS-CoV-2 suspended in universal transport medium.

Amid the pandemic, we have witnessed rapid progress in the development of different diagnostic kits for COVID-19. However, the race continues and nanosensors are an integral part of this process in the search for new solutions and have contributed significantly to the process of translating in vitro systems into in vivo systems [119]. The effect of the corona protein, which is when the surface of nanoparticles in the medium of biological fluids is quickly covered by a selected group of biomolecules, has been extensively investigated for these nanosensors. When functionalized with the appropriate receptors, nanoparticles can act by specifically recruiting viral proteins during the formation of corona proteins [120].

Therefore, progress in research related to nanosensors depends on a detection system that is ultra-sensitive and can combine low-cost, high-speed and simple instrumentation. In this context, the future for these nanotechnology-based systems is to explore the integration of various properties (optical, magnetic, electrochemical and biological) to promote a more precise and fast response for the diagnosis [121]. As demonstrated throughout this topic, nanotechnology has been extensively investigated in the development of new detection systems. Table 3 provides an overview of the sensors developed for the detection of SARS-CoV-2, which is responsible for COVID-19.

**Table 3:** Nanotechnology-based sensors for detection of SARS-CoV-2 (COVID-19)

Sensor type	Viruses	Analytical data	Samples	Detection speed	Interferents	Detection medium	References
Polymeric nanoparticles coated with streptavidin dye	SARS-CoV-2	LOD: 12 copies Sensitivity: 100% (33/33) Specificity: 100% (96/96)	Oropharynx swab	60 min for the whole diagnosis	Absence of cross reactions	Colorimetric	[122]
Lanthanide-doped polysterene nanoparticles	SARS-CoV-2	Was used to test seven samples that were positive by RT-PCR and 12 that were negative Results of this assay: 8 positive and 11 negative	Blood serum	10 min	–	Fluorimetric	[123]
Poly (amino ester) with carboxyl groups coated magnetic nanoparticles (pcMNP)	SARS-CoV-2	LOD: 10 copies Linear range: 10–10^5^ copies	Pseudovirus samples diluted in foetal calf serum	20 min for purification + subsequent RT-PCR reactions	Some signal amplifications in negative controls, although about 40 delayed cycles of a valid positive result	Direct RT-PCR	[116]
Gold nanoparticles	SARS-CoV-2	Sensitivity: 88.66% (352/397) Specificity: 90.63% (116/128)	Venous blood and finger prick	15 min	–	Colorimetric	[114]
Gold nanoislands	SARS-CoV-2	LOD: 0.22 pmol/L Linear range: 0.1 pmol/L–1 μmol/L Recovery rate: 26/5000 96% in mixing sample.	Synthetic oligonucleotides	–	–	Interferometry	[124]
Zinc ferrite nanoparticles	SARS-CoV-2	–	–	15 min Extract the viral RNA through automation process + subsequent RT-PCR reactions	–	Direct RT-PCR	[125]
Nanopore target sequencing (NTS)	SARS-CoV-2 and other respiratory viruses simultaneously	LOD: 10 copies Linear range: 10–3000 copies Specificity: 100% (5/5)	Oropharyngeal swabs	6–10 h	–	qPCR	[126]
Spike (S) protein specific nanoplasmonic resonance sensor	SARS-CoV-2	Linear range: 10^3^ virus particle/mL–10^6^ virus particle/mL LOD: 750 vp/mL	Pseudovirus diluted in bovine serum albumin (BSA)	15 min	–	Colorimetric	[127]

## Carriers and drug delivery systems with potential to control viral infection

With the pandemic caused by SARS-CoV-2, it is necessary to search for strategies to contain this new and deadly human infection. Several drugs employed in the treatment of other diseases have been suggested as possible inhibitors of SARS-CoV-2; however, some of them can cause serious side effects or are still in the testing phase [128, 129], and until now, there are none with proven efficacy. The antiviral substances considered to contain SARS-CoV-2 have, as their potential mechanism of action, an aim to change or inactivate viral surface proteins, such as the spike glycoprotein, that is responsible for their binding and entry into cells [130, 131] or act in the inhibition of viral replication [132]. Although investigations regarding the efficacy of these and other drugs are in progress, it is important to consider that many of these substances have specific actions [130, 133], which makes them prone to loss of effectiveness as a result of possible viral mutations and development of resistance [67–69]. There is some evidence from analysing patient samples that SARS-CoV-2 is mutating, which may make available drugs ineffective [120]. In addition, some drugs are only effective in high concentrations, which can cause toxicity to host cells and consequently side effects [128, 134].

In addition, viruses belong to the same family and share similar characteristics, which makes it possible to use drugs already approved by the FDA for the treatment of emerging viruses. Viral epidemics are challenging from a therapeutic and public health point of view. According to Clercq and Li [135], there are 90 antiviral active drugs approved for the treatment of viral infections. Nevertheless, the administration of these drugs is often accompanied by side effects, and most of them are poorly water-soluble, which limits their successful use. For example, chloroquine and hydroxychloroquine have been associated with cardiotoxicity, as well as hepatotoxicity and nephrotoxicity [136], while ribavirin is associated to haemolytic anaemia [137]. Most side effects of antiviral drugs are generated due their accumulation in off-target organs. In this way, approaches that could target the delivery of the drug into the desired organ and/or decrease the toxicity of these drugs are very promising to improve the efficacy of antiviral treatment [28].

It is already known that nanocarriers are able to change the pharmacokinetic parameters of the encapsulated drug and decrease drug concentration required for biological activity due to sustained and/or controlled release [67]. In addition, the application of target ligands on the surface of nanocarriers for recognition of molecular components of the tissue/organ of interest is a very promising approach to boost antiviral effects [138]. In summary, the association of the abovementioned FDA approved drugs with nano-based carriers seems to be revenue to create highly effective antiviral formulations and decrease the side effects and toxicity of conventional treatments of viral infections. In addition, it will be possible to decrease the speed of the development of resistance through the encapsulation of these drugs [28, 29].

A study performed by Leuschner et al. brings a direction in the use of nanotechnology to control the cytokine storm. Cytokine storm is one of the clinical complications of COVID-19. It consists of the exacerbated production of pro-inflammatory cytokines, which leads to dysfunction in multiple organs and rapid clinical deterioration [139, 140]. The produced small interfering RNA (siRNA) encapsulated in lipidic nanoparticles are specific for silencing the chemokine receptor CCR2, which inflammatory monocytes depend on to find areas of inflammation. Adequate degradation of CCR2 messenger RNA in monocytes avoids its accumulation in inflammatory sites. In this way, the uncontrolled recruitment of monocytes in inflammatory processes was solved, with promising results obtained in mice [141]. It is already known that some commercially available medicines, such as tocilizumab and C_1_ esterase inhibitor, have shown positive results in the control of cytokine storm; however, these are very expensive drugs, and their production is not easily scalable. In order to both reduce the treatment costs and streamline the production, Testori suggests the use of plasmids as engineered vectors to produce interleukin (IL)-10 inside cells to contain the inflammatory process [142]. Nanoparticles made of polyethylenimine (PEI) [143], PLGA [144] PEG-PCL [145], PLA-PEG [146] and so on have been successfully studied with regard to the systematic delivery of interleukins as immunotherapy for different types of diseases, and these findings could be helpful in the development of efficient nanoparticle therapies for cytokine storm in patients with COVID-19.

Dormont et al. used squalene, an endogenous lipid for preparing nanoparticles loaded with the anti-inflammatory drugs adenosine and alpha-tocopherol (vitamin E), for targeted action in regions of acute inflammation [147]. Mice in an acute hyperinflammatory state and with cytokine storm were treated with squalene nanoparticles, and a reduction in the levels of pro-inflammatory cytokines and an increase in the levels of IL-10 were observed, mitigating uncontrolled inflammation. In addition, it was observed that the encapsulation of adenosine increased its half-life and consequently increased its therapeutic effect when compared to non-encapsulated drugs. Due to the targeted delivery of adenosine to the loci of inflammation, combined with the ability of decreased reactive oxygen species at the inflammatory site, this nano-based formulation holds promise as a treatment for uncontrolled inflammation caused by coronavirus.

Also, curcumin has shown antiviral activity against several viral infections, including hepatitis, influenza, Zika virus, chikungunya virus and other sexually transmitted viruses. Just recently, Loutfy et al. synthesized chitosan nanoparticles loading curcumin against hepatitis C virus genotype 4a. Chitosan nanoparticles were able to inhibit 100% of viral infection and replication in human hepatoblastoma cells (Huh7) [148]. The antiviral activity of nanoparticles containing curcumin was due to disturbances in the fluidity of the virion membrane, but no changes in virion integrity were observed. Nanoparticles were able to inhibit both virus entry into the hepatoblastoma cell and replication. Curcumin is just one example within the wide range of natural compounds that can potentially be used to control viral infections, including COVID-19.

Therefore, the use of nano-based formulations has indicated a great potential for the control of viral infections, where nanoparticles can both enhance the efficacy of an antiviral drug and also reduce its toxicity [29]. Nanotechnology has also been used to enhance the efficacy of antiviral drugs by overcoming their low bioavailability. The development of nanomaterials, such as nanogels, which can capture the viable virus particles or viral RNA/proteins, are also promising developments that can help in the fight against SARS-CoV-2 [149, 150]. The main goal of future research of nano-based antiviral therapies will be the development of nano-based formulations that can successfully target precise sites of viral infection (e.g. the respiratory system in the case of COVID-19), reduce drug toxicity in other tissues, and potentially have some intrinsic antiviral activity of its own.

### Nanoparticles design for virus inhibition

Nanomaterials in the nanometre range (i.e. smaller or close to the size of viruses) may be combined with active antiviral substances to improve the bioavailability and interaction of the latter with the viral particles. The activity of some nanomaterials (e.g. silver and gold nanoparticles) may also contribute towards the overall antiviral action. An appropriately designed nanoparticle-antiviral drug combination can be expected to enhance the effect of the compounds in several ways (e.g. facilitate interaction with the viral particles, interfere with their entry into cells, increase bioavailability and stability of the formulation, and release antiviral agents in a controlled manner) [67, 151, 152]. Biocompatible nanoparticles may show broad-spectrum antiviral activity [153].

**Fig. 5:**
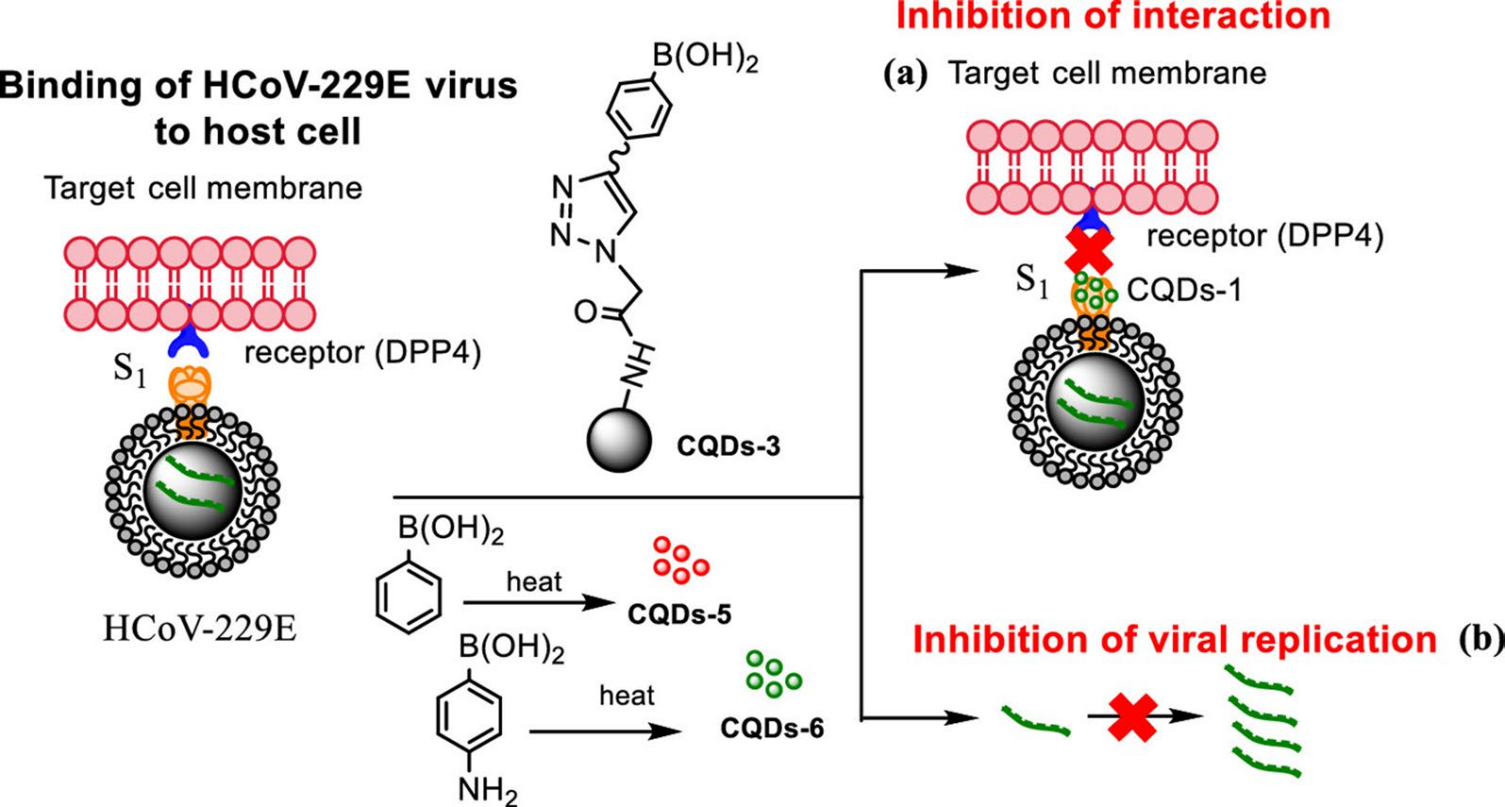
Influence of CQDs, prepared by hydrothermal carbonization, on binding of HCoV-229E virus to cells: **a** inhibition of protein S receptor interaction, and **b** inhibition of viral RNA genome replication (Reprinted with permission from Loczechin et al. [151])

Nanomaterials can be designed to have different functional groups on the surface and to bond with specific cell receptors, and these approaches can be used to block the contact of the virus with target cells. Loczechin et al. used carbon quantum dots (CQDs) combined with boric acid to inhibit human coronavirus HCoV-229E (Fig. 5). In this study, the interaction of the functional groups (boronic acid) of the CQDs with the receptors of the virus and with glycoprotein S was observed, interfering in the binding of the virus with the cell [151]. According to the authors, the CQDs inhibit the infection either by interaction with the S protein of HCoV-229E or by interaction with entry factors. In addition, CQDs were able to inhibit the virus in the replication step. In this way, this kind of approach could be useful for the inhibition of an infection with SARS-CoV-2 at different stages of the infection.

Focusing on the cell surface receptors heparan sulphate proteoglycans (HSPGs), which are the binding regions of some viruses in cells, including coronaviruses, Baram-Pinto et al. applied gold nanoparticles capped with mercaptoethanesulfonate (Au-MES NPs) to mimic this receptor, aiming to inhibit herpes simplex virus type 1 (HSV-1) attachment to cells [154]. This strategy interferes with the attachment and entry of the virus, in addition to its spread from one cell to another. Although this study was carried out with the herpes virus, this tool can also be explored for the inhibition of SARS-CoV-2, considering that HSPGs are one of the pathways for the entry of this virus into cells [155]. In a similar study, Cagno et al. synthesized gold nanoparticles coated with undecane sulfonic acid mimicking HSPGs. The authors reported that the virus associates with these structures and undergoes deformation, losing its infectious potential. The nanoparticles caused the definitive inhibition of different viruses, such as HSV, human papilloma virus (HPV), respiratory syncytial virus (RSV), dengue and lentivirus in vitro, HSV-2 ex vivo and RSV in mice, which indicates a broad-spectrum potential [156]. As in the previous study, with the aim of mimicking heparan sulphate, Jones et al. developed cyclodextrins modified with mercaptoundecane sulfonic acids, which promoted broad-spectrum antiviral activity in vitro against different HSPG-dependent viruses, with good compatibility and using low concentrations. In addition, promising results were also observed in ex vivo and in vivo assays, with no report of toxicity or development of resistance [157].

In another strategy, siRNA also appear as a promising tool for the control of SARS-CoV-2. In 2005, Wu et al. evaluated the effectiveness of siRNA targeted to the coding sequences of the S protein of SARS-CoV-1, which is responsible for the pathogenicity of the virus, and the 3′-UTR region and obtained inhibitory effects on the replication of the virus in the Vero E6 cell line [158]. Using the same strategy, Li et al. showed interesting results for the control of SARS-CoV-1 using siRNA [159]. Gene expression and viral replication were inhibited in Vero E6 cells employing siRNAs, which were prepared targeting a conserved leader sequence common to all coronaviruses. Considering the existing similarities between SARS-CoV-1 and SARS-CoV-2 [160, 161], these alternatives may be considered in the search for solutions against COVID-19. In a letter to the editor of EXCLI Journal, Ghosh et al. proposed the use of siRNA for the treatment of COVID-19 [162].

They indicate the highly conserved coding sequence for 3-chymotrypsin-like protease (nsp5) and the RNA polymerase-dependent viral RNA as targets for siRNA. However, due to its high molecular weight (~ 13 kDa), negative charge, easy degradation by enzymes and difficulty crossing cell membranes, naked siRNA could not reach the target cell efficiently. To overcome these hurdles, a non-viral gene delivery system has been studied to efficiently deliver them to target cells and/or tissues [163].

### Nanoparticles as carrier systems for clustered regularly interspaced short palindromic repeats (CRISPR)

CRISPR is a gene editing technique with high precision and potential to be used in gene therapy. Mainly, CRISPR/Cas9 are inserted into the cells in the form of plasmids, mRNA or ribonucleotide proteins, which can be used by nanocarrier systems to increase the transfection of cells [164, 165].

In addition, CRISPR-based systems have also been highlighted as an alternative to be explored in the search for containment of SARS-CoV-2. Abbot et al. developed a strategy based on CRISPR against SARS-CoV-2 and influenza, denominated PAC-MAN (prophylactic antiviral CRISPR in human cells) [153]. Cas13d RNA endonuclease along with guide RNA were used to inhibit and degrade the viral genome and synthesize mRNA in a targeted manner. Promising results were obtained, with degradation of the RNA of the sequences of SARS-CoV-2 and influenza A virus in A549 cells (human lung epithelium).

Nguyen et al. also consider the CRISPR/Cas13d system as an alternative for the control of SARS-CoV2. They developed 10,333 guide RNAs targeting 10 virus peptides and used adeno-associated virus (AAV) for the targeted transport of cas13d and guide RNAs to the lung. According to the authors, this is an innovative and fast approach [120]. Tanaka et al. showed a perspective for the treatment of COVID-19 from the edition of a CRISPR/Cas9 system using protein modelling tools. The system, employing specific guide RNA and a specific single-stranded oligodideoxynucleotide (ssODN), induced point mutations in the region of the human ACE2 gene involved in binding of the S protein of SARS-CoV-2, weakening that [166]. As ACE2 is also involved in the conversion of angiotensin 2 to control blood pressure, the conformational structure of this region has been preserved so as not to cause deleterious effects on its functions.

Although CRISPR-based solutions are very promising for the control of COVID-19, there is still a limitation on finding the most appropriate delivery systems. Some challenges are: (i) Cas9 proteins have a molecular weight around 150 kDa and a positive charge, which can decrease its encapsulation by nanoparticles; (ii) the nanoparticles need to cross the cell’s nucleus and release CRISPR/Cas9 for effective gene editing; and (iii) CRISPR/Cas9 are derived from bacteria that can activate the immune system; for this reason, a nanocarrier system that avoids triggering the host’s immune system needs to be developed [167]. Viral vectors (e.g. AAV) are the most common type of delivery; however, they possess some limitations such as small insertion size, high carcinogenesis risk, and immune system stimulation [168, 169]. Nanotechnology can contribute with new delivery alternatives for the exploitation of CRISPR-based systems. As an example, Lee et al. (2017) developed a delivery system for Cas9 ribonucleoprotein and donor DNA based on functionalized gold nanoparticles and obtained interesting results in the correction of DNA mutation in vitro and in mice afflicted by Duchenne muscular dystrophy [170]. Many recent studies have focused on the development of delivery systems for CRISPR and have obtained promising results [167–169, 171, 172], making this an approach to be explored for the application of these systems against COVID-19.

## Development of vaccines

Vaccination has been one the most effective public health programmes ever announced to prevent and/or control the spread of contagious diseases, by using the intrinsic ability of the immune system to induce protective long-term immune memory. Amongst all the components of vaccines, there are two key components: an antigen, which is the target of the immune response, and an adjuvant, which is a co-administered substance responsible for potentiating and/or modulating the immune response against the antigen [173].

So far, there are three different generations of vaccine formulations used to elicit immunological responses against infectious diseases, including attenuated or inactivated whole pathogen (first generation), recombinant subunit vaccines (second generation) and RNA or DNA vaccines (third generation) [174]. Live attenuated or inactivated whole pathogen vaccines have been extensively used to prevent and control diseases in humans and animals. However, the genetic reversion of attenuated vaccines or infection of immunocompromised hosts, as well as the likely tumorgenicity of deactivated viruses, are essential safety issues. In addition, all microorganisms cannot be used as live vaccines, since some of them are extremely virulent and others are intrinsically immune evasive [175, 176].

As alternative to live attenuated or inactivated vaccines, the second generation of vaccines were developed, based on non-pathogenic resources, which could be synthetic peptides, inactivated toxins or recombinant subunit protein vaccines. However, some limitations of these types of vaccines are the poor immunogenicity of the antigens, needing an adjuvant to enhance its immune response. In addition, premature degradation of the antigen in hostile environments hinders the effectiveness of vaccines. Finally, so far, the most modern vaccines are based on DNA or RNA, which also have some disadvantages, such as failing to reach the target sites and the necessity of a prime-boost vaccination scheme with other immunogenic agents, as well as premature degradation of the antigen, which results in weak immune response [177]. Despite it being promising, it is worth mentioning that this type of vaccine is not available on the market.

### Nano-based vaccines

Recently, nanoparticles have caught attention as a promising approach to the development of a new generation of vaccines, since the nanoparticles can both serve as a carrier for the antigen and behave as an adjuvant in many cases. In addition, nano-based vaccines can protect antigens against premature degradation and provide sustained release, enhanced antigen stability, and provide targeted delivery of an immunogen, as well as increase the period of antigen exposure and uptake by antigen presenting cells (APCs) [42, 175]. Furthermore, nanoparticles are able to interact with immune machineries, inducing cellular and humoral immunological responses. Studies have shown that nanoparticles who range in size between 20 and 200 nm are preferentially internalized by endocytosis into APCs (resulting in the T cell response), while large particles (0.5-5 µm) are usually internalized by phagocytosis (inducing the humoral immune response) [178, 179].

The nanoparticles work on the same scale as viruses and can be designed to release a compound into a specific target. The fact that these systems can be developed to cross cell membranes and target specific subcellular locations enhances the potential of nano-based vaccines. For this, different materials can be used for the development of nanocarriers, such as lipids, polymers and polysaccharides [180]. For instance, lipidic nanoparticles for the encapsulation of genetic material enhance the immune response to the vaccine, once the nanocarrier system can protect the DNA or RNA against enzymatic degradation and increase cell uptake, releasing the genetic material in target cells [181].

Antigens can either be encapsulated inside the nanocarriers or bound (conjugated) to the surface of the nanoparticle and administered together with the adjuvant to the target [42, 182]. To date, a large variety of delivery systems, such as polymeric nanoparticles, lipid nanoparticles, virus-like particles, virosomes, liposomes, emulsions, proteins and immune-stimulating complexes, have been investigated as antigen carriers [41, 183, 184]. The efficacy of vaccines may be further enhanced through targeted modifications to the nanoparticle-antigen conjugates to achieve the desired level of immunological response [41, 42]. Amongst these properties, the size, shape and surface charge of the nanoparticles can be controlled, as well as the functionalization of the surface with a variety of ligands to make them adaptable vehicles for vaccines [42, 185]. The route of administration could be via subcutaneous or intramuscular injection or by oral or intranasal mucosa, as well as capillary penetration [42, 186].

The development of a vaccine for the novel coronavirus can be challenging. Some studies have shown that the S protein is an excellent target for vaccine development; however, optimizing the design of the antigen is essential to ensure an adequate immune response [187, 188]. To date, studies have used the full-length S protein or carefully chosen regions of the protein, for example, the receptor-binding domain (RBD) or N-terminal domain, which are combined with adjuvants in order to enhance the immunological response [189]. Besides the S protein, other antigens such as non-structural proteins and nucleoproteins seem to be good candidates for the development of “cocktail” vaccines against SARS-CoV-2 [190]. This approach has been investigated by Epivax, which is working on a cocktail vaccine aiming to generate at least partial protection against SARS-CoV-2 while waiting for more efficient vaccines to be available [191]. Vaccine candidates formulated with the full-length S protein of SARS-CoV-2 combined with saponin-based Matrix-M™ adjuvant developed by Nanovax is currently in Phase 1/2 clinical trials (NCT04368988). It has been demonstrated that the Matrix-M™ adjuvant stimulates the entry of APCs into the injection site, which enhances the antigen presentation in local lymph nodes, increasing the immunological response [192, 193].

Different from subunit vaccines, RNA-based vaccines work by introducing a mRNA encoding a disease-specific antigen. Once the sequence of interest is inside the cells, it serves as a template to produce the antigen (protein) in situ. After translation, the antigen could be extracellularly transported and recognized by antibodies or could be intracellularly processed and presented to T-cells, resulting in the humoral and cellular immune response, respectively [194, 195]. On the vaccine front, Moderna, in collaboration with Vaccine Research Center at the U.S. National Institutes of Health, has developed mRNA vaccines (mRNA-1273) encapsulated in lipid-based nanoparticles. The vaccine is currently in a Phase II clinical trial, which has the participation of 600 healthy individuals (NCT04405076).

Vaccines candidates for COVID-19 were developed right after the publication of the complete genome of SARS-CoV-2. According to a report produced by the WHO, up to 9 June 2020, there were 136 vaccine candidates for COVID-19 being developed, 10 of which are currently in clinical trial phases [196]. In addition, 16 are nano-based vaccines, which are currently under R&D for prevention of COVID-19. The technology used in nano-vaccine candidates are summarized in Table 4. With the understanding of the interactions between nanoparticles and the immune system, it can be expected that nanotechnology will fare better in terms of delivering quicker, safer and more effective vaccines compared to those developed by conventional approaches.

**Table 4:** Nano-based vaccine candidates to prevent COVID-19 infection

Name	Developer	Method/platform	Development phase
Moderna coronavirus vaccine	National Institutes of Health (NIH) and Moderna (United States)	mRNA-based vaccine, which encodes the full-length of the spike (S) protein encapsulated in lipid nanoparticles	Phase 1 NCT04283461 Phase 2 NCT04405076
NVX-CoV2373	Novavax, Inc. (United States)	Virus-like nanoparticle, which contains SARS-CoV-2 S protein combined with adjuvant matrix -M	Pre-clinical
Ad5-nCoV	Cansino Biologics, Inc. (China)	Adenovirus 5 vector, which contains SARS-CoV-2 S nanoparticles produced in the baculovirus insect cell expression system	Phase I NCT04313127
COVID-19 vaccine candidate	BioNTech/Fosun Pharma/Pfizer (Germany)	Lipid-based nanoparticles (LNPs) combined with mRNA.	Phase I/II NCT04368728
COVID-19 vaccine candidate	Viroclinics Xplore (Netherlands)	UQ`S molecular clamp technology, which locks the S protein conformation to mimic the protein found on the live virus	Pre-clinical
COVID-19 vaccine candidate	Ufovax, LLC (United States)	Virus-like particle with features of SARS-CoV-2 S protein protruding from a protein nanoparticle scaffold	Pre-clinical
COVID-19 vaccine candidate	Janssen Pharmaceuticals, Inc. (Belgium)	Recombinant vaccine using AdVac^®^ technology, which is based on the development and production of adenovirus vectors (gene carriers) combined with the PER.C6^®^ cell line	Pre-clinical
COVID-19 vaccine candidate	Translate Bio/Sanofi Pasteur (United States)	LNPs loading mRNA encoding functional proteins from SARS-CoV-2	Pre-clinical
DPX-COVID-19	IMV, Inc. (Canada)	LNPs formulated with DPX platform, containing peptides epitopes from SARS-CoV-2 S protein	Pre-clinical
COVID-19 vaccine candidate	CanSino Biologics/Precision NanoSystems (China/Canada)	LNPs combined with mRNA	Pre-clinical
COVID-19 vaccine candidate	Fudan University/Shanghai JiaoTong University/RNACure Biopharma (China)	LNPs loading mRNA encoding the receptor-binding domain of SARS-CoV-2 S protein	Pre-clinical
COVID-19 vaccine candidate	Fudan University/Shanghai JiaoTong University/RNACure Biopharma (China)	LNPs loading mRNA that induces the formation of virus-like particles similar to native SARS-CoV-2 in the host	Pre-clinical
COVID-19 vaccine candidate	University of Tokyo/Daiichi-Sankyo (Japan)	LNPs combined with mRNA	Pre-clinical
COVID-19 vaccine candidate	BIOCAD (Russia)	LNPs formulated with recombinant vesicular stomatitis virus (rVSV) that expresses mRNA from SARS-CoV-2	Pre-clinical
COVID-19 vaccine candidate	St. Petersburg Scientific Research Institute of Vaccines and Serums (Russia)	LNPs formulated with recombinant S protein and other epitopes from SARS-CoV-2	Pre-clinical
COVID-19 vaccine candidate	LakePharma, Inc. (United States)	Recombinant vaccine containing COVID-19 S proteins created using CHO manufacturing platforms	Pre-clinical

## Conclusions and perspectives

Very little is known about SARS-CoV-2, and therefore, there are currently more questions than answers. Research addressing the aetiology, epidemiology, mechanism of pathogenesis and detailed host immune response to the virus needs to work together to develop diagnostics, treatments and other control measures to combat the epidemic. Recent research into the use of metal nanoparticles as antimicrobial agents [197–199] can provide new solutions for surface decontamination and enhanced efficacy of PPE products used by healthcare workers [64]. Nanotechnology has already been employed in the diagnosis and treatment of other viral diseases and may provide a “fresh start” for trying pre-existing drugs and treatments against COVID-19, by addressing the issues of toxicity, poor stability and low bioavailability [200]. Furthermore, nano-based formulations may decrease the development of antiviral resistance, which is a common problem for many conventional antiviral drugs currently available [28]. Nano-based formulations could also be designed to target a specific tissue and with controlled-release properties, which would increase the efficiency of treatment and consequently reduce the period and dose of the treatment for control of the virus. Altogether, these approaches could simplify the multidrug therapies that are currently used to treat infectious diseases [106].

Nanotechnology applications, however, have some bottlenecks that need to be addressed to facilitate its broader implementation in the wider healthcare system. One of the major challenges is to ensure the safe use of nanomaterials, since most of the studies have only evaluated the biocompatibility using in vitro approaches. The fate and behaviour of nanomaterials in the body can also change when they reach blood circulation due to the formation of protein corona [201]. Thus, reliable in vivo models are needed to better understand the toxicokinetic behaviour of the nanoparticles in the body, especially for long-term exposure [202]. Another issue is the lack of standardized protocols for physicochemical and biological characterization of nanomaterials, as well as lack of a universally agreed upon definition of a nanomaterial [203]. Due to these limitations, generic protocols have been employed for characterization during the early stages of R&D, which explains the huge numbers of failures in terms of clinical translation of the final nano-based therapies [204]. To overcome the abovementioned hurdles, a closer collaboration between regulatory agencies, scientific experts in material science, pharmacology and toxicology is needed. Capacity for large-scale manufacturing is another hurdle that needs to be overcome for broader commercialization of nano-based formulations [32, 205].

Due to the multifaceted interactions between nanomaterials and biological systems (in vivo), it is very challenging to foresee the behaviour of these materials under physiological conditions. Once within the body, the nanoparticles reach the blood circulation, which is a very complex matrix containing ions, small molecules, proteins, and cells [206]. It is already known that nanoparticles are able to interact with biomolecules, mainly proteins, resulting in the formation of protein corona [207]. The composition of protein corona is mainly driven by the physicochemical properties of the nanoparticles. In other words, it is unique for each nanoparticulate system and influenced by several factors. Both protein-nanoparticle interaction and protein–protein interaction regulate the adsorption of protein on the surfaces of nanoparticles [207]. The formation of protein corona modifies the physicochemical properties of nanoparticles, consequently giving them a new biological identity, which is more significant in determining the biological response than the original properties of the nanoparticles [208]. Therefore, the characterization of protein corona is an essential step to be investigated in the process of nanomedicine development.

In conclusion, as this review shows, nanotechnology has already been shown to enhance diagnostics, protection and therapies in other viral infections; therefore, there is a good chance that, with more R&D, it will revolutionize the fight against COVID-19 (and any other future outbreaks), offering processes, materials and tools to enhance sensitivity, speed and reliability of diagnosis, as well as providing more efficacious options for therapies. However, as researchers working with nanotechnology, we believe that only through effective and close collaboration between the different society stakeholders will we be able to respond quickly to any future global health emergencies. Therefore, it is extremely important that research centres, universities, commercial companies, and the medical community, as well as regulatory agencies and the government, combine efforts to streamline the use of these new tools and technologies for the benefit and protection of society. We also emphasize the need for investment in scientific research, both through public and private funding. This will make it possible to produce and transform knowledge into products, which, in addition to combating the current pandemic, will also provide means for prevention of future outbreaks.

## Data Availability

Not applicable.
